# The Integrative and Conjugative Element ICE*Csp*POL2 Contributes to the Outbreak of Multi-Antibiotic-Resistant Bacteria for *Chryseobacterium* Spp. and *Elizabethkingia* Spp.

**DOI:** 10.1128/Spectrum.02005-21

**Published:** 2021-12-22

**Authors:** Jiafang Fu, Chuanqing Zhong, Yingping Zhou, Mengru Lu, Gongli Zong, Peipei Zhang, Moutai Cheng, Guangxiang Cao

**Affiliations:** a Department of Epidemiology, The First Affiliated Hospital of Shandong First Medical University, Jinan, China; b College of Biomedical Sciences, Shandong First Medical University & Shandong Academy of Medical Sciences, Jinan, China; c School of Municipal and Environmental Engineering, Shandong Jianzhu University, Jinan, China; Universidad de Buenos Aires, Facultad de Farmacia y Bioquimica

**Keywords:** *Elizabethkingia*, antibiotic resistance genes, *Chryseobacterium*, ICE*Csp*POL2, horizontal gene transfer

## Abstract

Antibiotic resistance genes (ARGs) and horizontal transfer of ARGs among bacterial species in the environment can have serious clinical implications as such transfers can lead to disease outbreaks from multidrug-resistant (MDR) bacteria. Infections due to antibiotic-resistant *Chryseobacterium* and *Elizabethkingia* in intensive care units have been increasing in recent years. In this study, the multi-antibiotic-resistant strain *Chryseobacterium* sp. POL2 was isolated from the wastewater of a livestock farm. Whole-genome sequencing and annotation revealed that the POL2 genome encodes dozens of ARGs. The integrative and conjugative element (ICE) ICE*Csp*POL2, which encodes ARGs associated with four types of antibiotics, including carbapenem, was identified in the POL2 genome, and phylogenetic affiliation analysis suggested that ICE*Csp*POL2 evolved from related ICE*Ea*s of *Elizabethkingia* spp. Conjugation assays verified that ICE*Csp*POL2 can horizontally transfer to *Elizabethkingia* species, suggesting that ICE*Csp*POL2 contributes to the dissemination of multiple ARGs among *Chryseobacterium* spp. and *Elizabethkingia* spp. Because *Elizabethkingia* spp. is associated with clinically significant infections and high mortality, there would be challenges to clinical treatment if these bacteria acquire ICE*Csp*POL2 with its multiple ARGs, especially the carbapenem resistance gene. Therefore, the results of this study support the need for monitoring the dissemination of this type of ICE in *Chryseobacterium* and *Elizabethkingia* strains to prevent further outbreaks of MDR bacteria.

**IMPORTANCE** Infections with multiple antibiotic-resistant *Chryseobacterium* and *Elizabethkingia* in intensive care units have been increasing in recent years. In this study, the mobile integrative and conjugative element ICE*Csp*POL2, which was associated with the transmission of a carbapenem resistance gene, was identified in the genome of the multi-antibiotic-resistant strain *Chryseobacterium* sp. POL2. ICE*Csp*POL2 is closely related to the ICE*Ea*s from *Elizabethkingia* species, and ICE*Csp*POL2 can horizontally transfer to *Elizabethkingia* species with the *tRNA-Glu-TTC* gene as the insertion site. Because *Elizabethkingia* species are associated with clinically significant infections and high mortality, the ability of ICE*Csp*POL2 to transfer carbapenem resistance from environmental strains of *Chryseobacterium* to *Elizabethkingia* is of clinical concern.

## INTRODUCTION

Antibiotic resistance has become a serious threat to human health (1, 2), and antibiotic resistance genes (ARGs) are considered a type of genetic pollution in the environment (3). As such, the effects of antibiotics on the environment in terms of ARGs, antibiotic-resistant bacteria, and horizontal transfer of ARGs have become an area of interest in the environmental sciences. There are four ways for bacteria to obtain ARGs: transformation (4), transduction (5), conjugation (6), and potentially through the fusion of two cells or the fusion of cells with DNA-containing vesicles (7). Of these four ways, conjugation is the more common method of horizontal gene transfer among bacteria in the environment. A study of 1,124 complete prokaryotic genomes revealed 180 putative conjugative plasmids and 335 putative integrative and conjugative elements (ICEs), suggesting that ICEs are likely more common than conjugative plasmids in prokaryotes (8).

ICEs are typically mosaic and modular genome-integrated mobile genetic elements, ranging from ∼20 kb to 500 kb in size, and are passively proliferated during genome replication, segregation, and cell division. Two typical features characterize ICEs: ICEs are integrated into a host genome, and they encode a type IV secretion/conjugation system, which enables them to transfer to other bacteria via conjugation (9–11). Many ICEs encode ARGs that confer antibiotic resistance on the recipient strain, and when the recipient strain is a human pathogen, this resistance can lead to difficulty in eradicating the pathogen and therefore potentially lead to outbreaks of infection. Elizabethkingia anophelis is an emerging opportunistic human pathogen that is associated with clinically significant infections and high mortality and has caused outbreaks in Singapore, Taiwan, Hong Kong, and the US state of Wisconsin (12, 13). An ICE named ICE*Ea*1 was identified in the Wisconsin outbreak strains and Singapore outbreak strains (12). Recently, three types of ICEs, ICE*Ea*I, ICE*Ea*II, and ICE*Ea*III, have been identified in pathogenic E. anophelis strains (13).

Livestock and poultry farms are commonly considered to be the major sources of ARGs. In this study, we isolated the multi-antibiotic-resistant strain *Chryseobacterium* sp. POL2 from a wastewater sample from a livestock farm in Shandong, China. In the chromosome of POL2, we identified a typical ICE, which we named ICE*Csp*POL2, that carries a carbapenem resistance gene. ICE*Csp*POL2 was closely related with ICE*Ea*s found in E. anopheles, and ICE*Csp*POL2 was found to horizontally transfer to *Elizabethkingia* sp., raising a warning regarding the need to track this kind of ICE in the environment to prevent further outbreaks of infection.

## RESULTS

### *Chryseobacterium* sp. POL2 is a multi-antibiotic-resistant strain.

After preliminary 16S rRNA gene analysis and then alignment of the whole-genome sequence, strain POL2 was identified as *Chryseobacterium* sp. MIC assays revealed that strain POL2 had some resistance to all tested antibiotics (Table 1), including ampicillin (MIC ≥32 mg/liter), cefixime (MIC ≥16 mg/liter), meropenem (MIC ≥64 mg/liter), amikacin (MIC ≥64 mg/liter), ciprofloxacin (MIC ≥128 mg/liter), tetracycline (MIC ≥128 mg/liter), florfenicol (MIC ≥128 mg/liter), sulfamethoxazole (MIC ≥128 mg/liter), vancomycin (MIC ≥32 mg/liter), and polymyxin E (MIC ≥16 mg/liter). Surprisingly, environmental isolate POL2 had striking resistance to meropenem, which is used in clinical disease treatment.

**TABLE 1:** Antibiotic susceptibility test of strain *Chryseobacterium* sp. POL2, *Elizabethkingia* sp. M6, and transconjugant *Elizabethkingia* sp. M6-P2

Antimicrobial agents	MIC (mg/liter)/antibiotic susceptibility^*a*^		
	POL2	M6	M6-P2
Ampicillin	32/R	72	96
Cefixime	16/R	<2/S	<2/S
Meropenem	64/R	8/R	36/R
Amikacin	64/R	16/R	48/R
Ciprofloxacin	>128/R^*b*^	>128/R	>128/R
Tetracycline	>128/R	32/R	72/R
Florfenicol	>128/R	16/R	96/R
Sulfamethoxazole	>128/R	32/R	32/R
Vancomycin	32/R	<2/S	<2/S
Polymyxin E	16/R	<2/S	<2/S

a Bacterial antibiotic susceptibility was interpreted according to the Clinical and Laboratory Standards Institute (CLSI) guidelines.

b R, resistant; S, susceptible.

### Genomic features and phylogenetic relationship of strain POL2.

**FIG 1:**
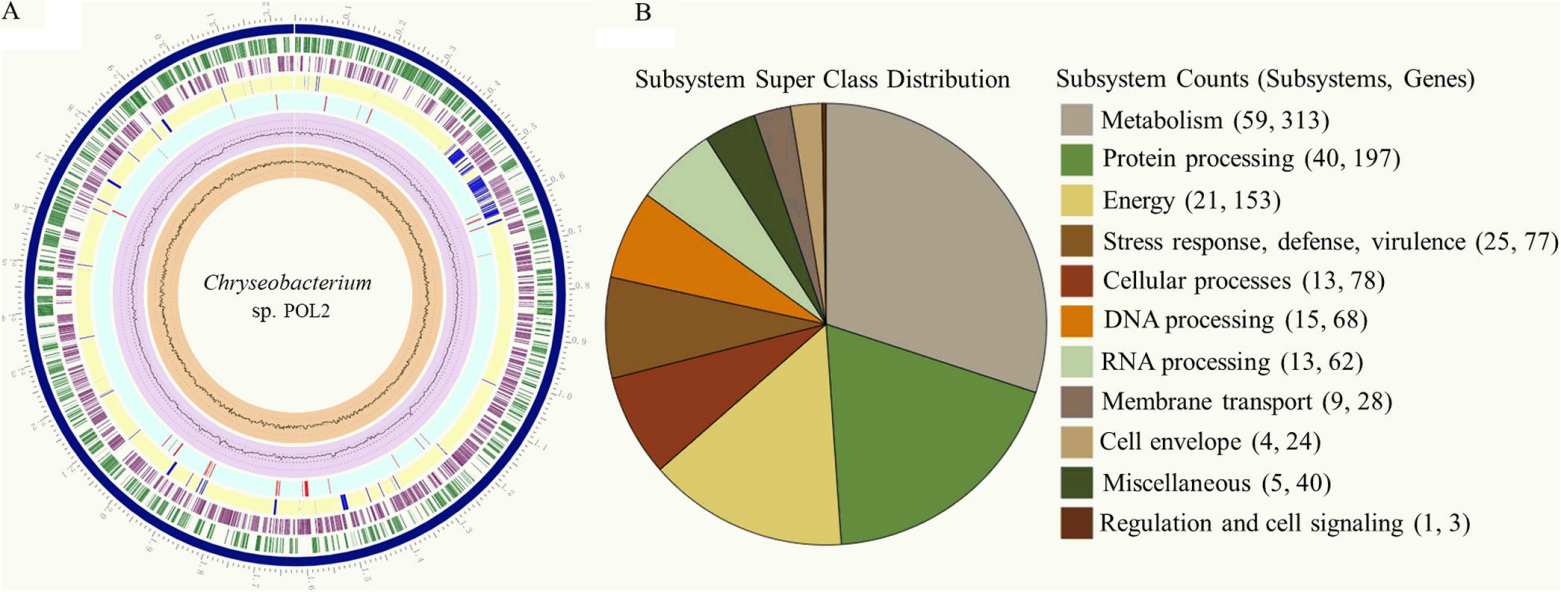
Comprehensive genomic analysis of *Chryseobacterium* sp. POL2. (A) Circular graphical display of genomic features. From inner to outer rings: GC skew; GC content; coding sequences (CDS) with homology to known antimicrobial resistance (AMR) genes; non-CDS features; CDS on the reverse strand; CDS on the forward strand; contigs/chromosome; and the position label (Mbp). (B) An overview of the subsystems/genes found in the *Chryseobacterium* sp. POL2 genome.

To understand the basis for its antibiotic resistance profile, the whole-genome sequence of strain POL2 was further analyzed (Fig. 1). POL2 contains only one circular chromosome, which is 3,243,462 bp in size, with an average GC content of 35.2%. The genome annotation revealed 3,003 genes in the POL2 genome, including 2,877 protein-coding genes, 15 rRNA genes, 49 tRNA genes, and three other RNA genes. Consistent with its antibiotic resistance profile (Table 1), genome annotation revealed that POL2 harbored dozens of antibiotic resistance-associated genes (Dataset S2), including genes coding for class D beta-lactamase, tetracycline-inactivating enzyme, and aminoglycoside 6-nucleotidyltransferase. In addition, a search for putative virulence genes led to the identification of 115 virulence factor-encoding genes (Dataset S3) based on the PHI database, indicating that POL2 is potentially pathogenic for humans and/or other organisms.

**FIG 2:**
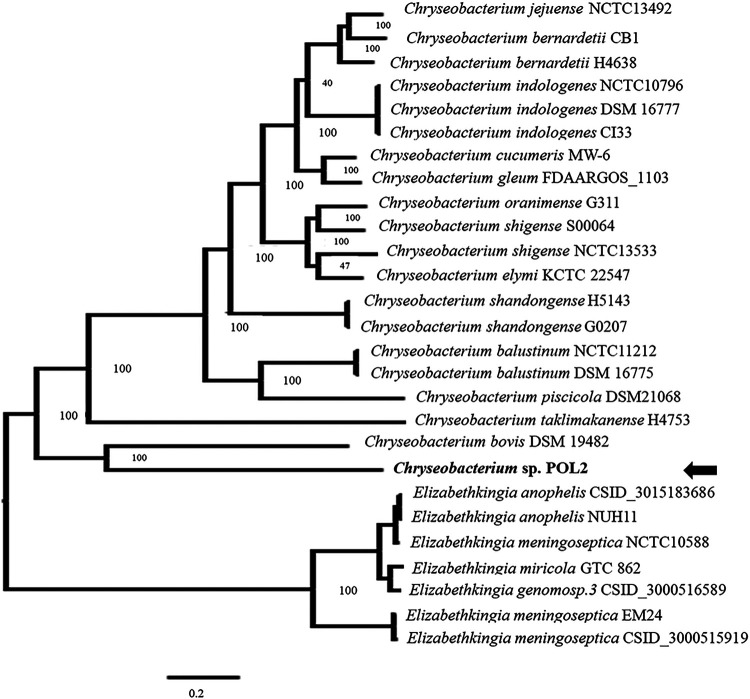
Molecular phylogenetic analysis of *Chryseobacterium* sp. POL2 based on the genome sequence. The whole-genome phylogenetic tree was constructed using the PATRIC server. The position of POL2 in the phylogenetic tree is indicated by the black arrow.

A phylogenetic tree analysis was also performed to determine the evolutionary relationship between POL2 and other *Chryseobacterium*/*Elizabethkingia* species (Fig. 2). The results revealed that strain POL2 is most closely related to strain Chryseobacterium bovis DSM 19482, although it is also related to *Elizabethkingia* spp., which like *Chryseobacterium*, belong to the *Flavobacteriaceae* family.

### Identification of the integrative and conjugative element ICE*Csp*POL2.

**FIG 3:**
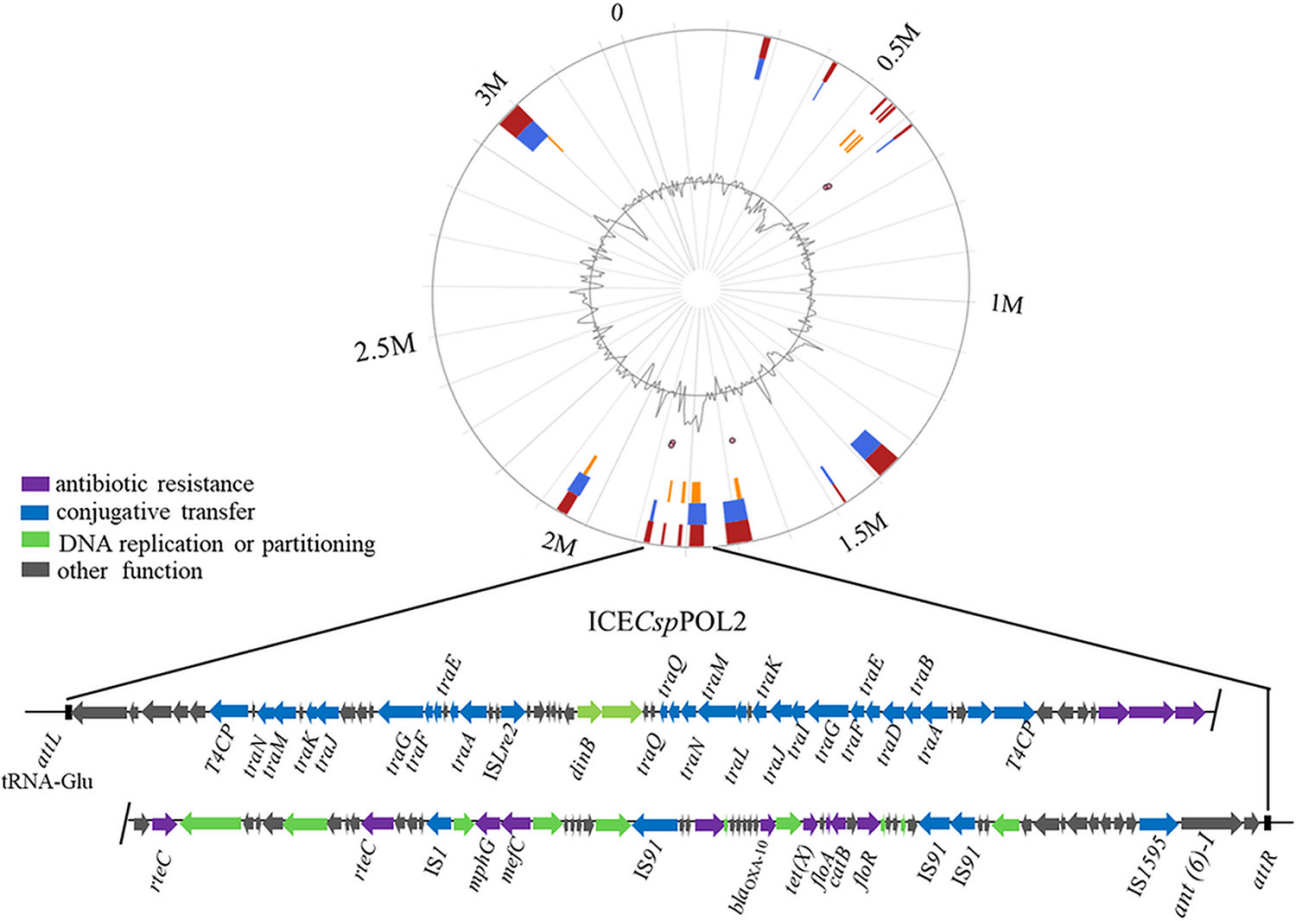
Schematic view of the integrative and conjugative element ICE*Csp*POL2. Top image: the predicted position of ICE*Csp*POL2 in the *Chryseobacterium* sp. POL2 genome using IslandViewer 4. Bottom image: gene arrangement and characteristics of ICE*Csp*POL2 identified using ICEberg 2.0. ICE*Csp*POL2 is bordered by an 18 bp DR (5′-ATTCCCCTACGGGCTACT-3′), indicated by black bars, in the chromosome of POL2. Violet arrows, ARGs; blue arrows, conjugative transfer genes; green arrows, DNA replication or partitioning genes; gray arrows, genes with other functions.

Preliminary analysis with the IslandViewer 4 program indicated that nucleotide positions 1,765,804 to 1,883,110 of the POL2 genome contained a potential antibiotic genomic island. Further analysis by ICEberg 2.0 verified a novel ICE in this region, which was named ICE*Csp*POL2 (Fig. 3). ICE*Csp*POL2 extends from position 1,767,025 to 1,883,295 in the POL2 genome and contains 116,271 bp. ICE*Csp*POL2 is bordered by an 18 bp direct repeat (DR) (5′-ATTCCCCTACGGGCTACT-3′) at both ends and is inserted into the 3′ end of the *tRNA-Glu-TTC* gene (G6R40_RS08190). Gene annotation revealed that ICE*Csp*POL2 contains 124 open reading frames (ORFs) (Table S2), including genes encoding conjugative elements, such as conjugative relaxases, ATPases of type IV secretion systems and the type IV coupling proteins, T4CP, and genes encoding DNA replication or partitioning-associated proteins and site-specific integrases. ICE*Csp*POL2 also contains ARGs, including two genes (*floR* and *catB*) associated with chloramphenicol/florfenicol resistance; two genes (*mphG* and *mefC*) associated with macrolide resistance; one gene (*tet*(X), encoding tetracycline-inactivating enzyme) associated with tetracycline resistance; one gene (*ant(6)-I*) associated with aminoglycoside resistance, and one gene encoding a Class D beta-lactamase OXA-10 like protein, which might be partially responsible for the meropenem resistance of strain POL2. Hence, ICE*Csp*POL2 has two typical characteristics of ICEs: it was integrated into the POL2 genome at the 3′ end of the *tRNA-Glu-TTC* gene and it encodes a type IV secretion/conjugation system.

### Phylogenetic relationship of ICE*Csp*POL2.

**FIG 4:**
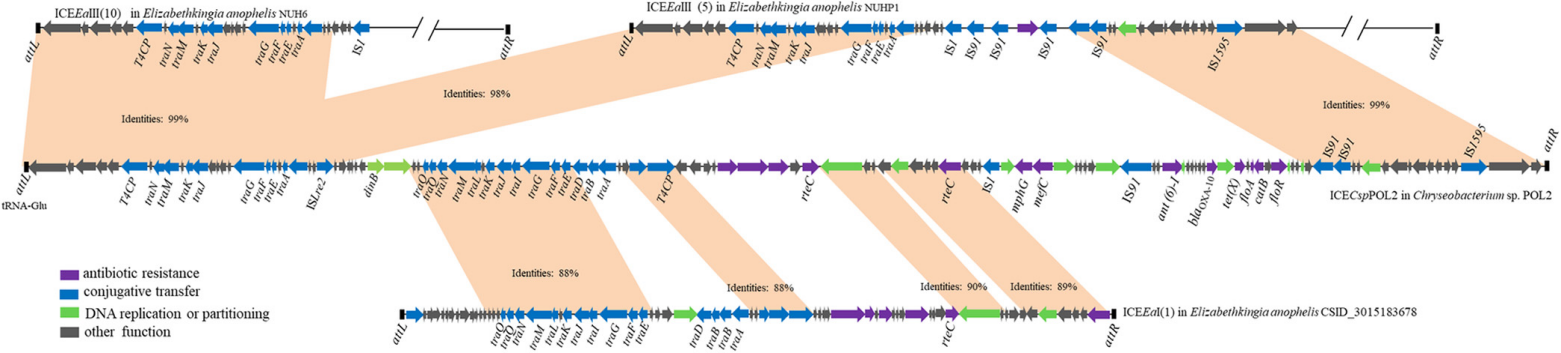
Schematic showing potential sources of genes in ICE*Csp*POL2. Gene sequences in ICE*Csp*POL2 of *Chryseobacterium* sp. POL2 were compared with gene sequences from ICE*Ea*III(10) of E. anophelis NUH6, ICE*Ea*III (5) of E. anophelis NUHP1, and ICE*Ea*I(1) of E. anophelis CSID_3015183678. Violet arrows, ARGs; blue arrows, conjugative transfer genes; green arrows, DNA replication or partitioning genes; gray arrows, genes with other functions. The 18 bp DR sequences are indicated by black bars.

The whole nucleotide sequence of ICE*Csp*POL2 was analyzed by BLAST, and pairwise alignment results revealed that ICE*Csp*POL2 has strong homology with the ICEs ICE*Ea*I and ICE*Ea*III in Elizabethkingia anophelis. Further BLASTn analysis revealed that genes in ICE*Csp*POL2 relevant to horizontal conjugative transfer were highly similar to genes in ICE*Ea*III(10) of the E. anophelis NUH6 strain; ICE*Ea*III (5) of the E. anophelis NUHP1 strain; and ICE*Ea*I(1) of the E. anophelis CSID_3015183678 strain (Fig. 4), indicating that ICE*Csp*POL2 likely evolved from related ICE*Ea*s of E. anophelis strains.

**FIG 5:**
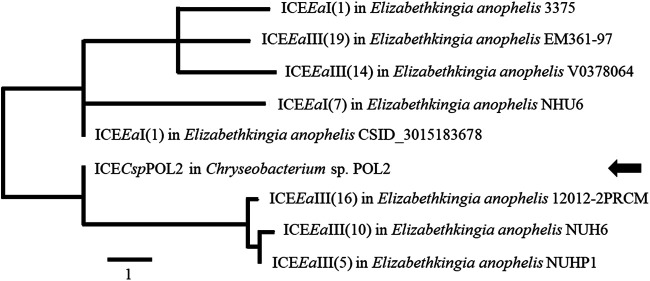
Phylogenetic relationships of ICE*Csp*POL2. Based on the ICE*Csp*POL2 whole nucleotide sequence alignment results, eight ICE sequences were selected, and the phylogenetic tree was constructed using MEGA7 software. The position of ICE*Csp*POL2 in the phylogenetic tree is indicated by the black arrow.

To further track the evolutionary history of ICE*Csp*POL2, eight related ICES of the ICE*Ea*s group were selected based on the BLASTn alignment results, and their nucleotide sequences were compared (Fig. 5). ICE*Csp*POL2 was most closely related to ICE*Ea*III(16) in E. anopheles 12012-2PRCM; ICE*Ea*III(10) in E. anopheles NUH6; and ICE*Ea*III(5) in E. anopheles NUHP1, with these ICEs forming a clade separate from ICE*Ea*I(1) in E. anopheles CSID_3015183678. Consistent with ICE*Csp*POL2, both ICE*Ea*III(10) and ICE*Ea*III(5) are also inserted into a *tRNA-Glu-TTC* gene (14), suggesting that these three ICEs are evolutionarily related to each other. Notably, ICE*Csp*POL2 harbors one Class D beta-lactamase gene, associated with carbapenem resistance, whereas the other reported ICE*Ea*s do not carry carbapenem resistance genes.

### Transfer of ICE*Csp*POL2 to another bacterial strain.

**FIG 6:**
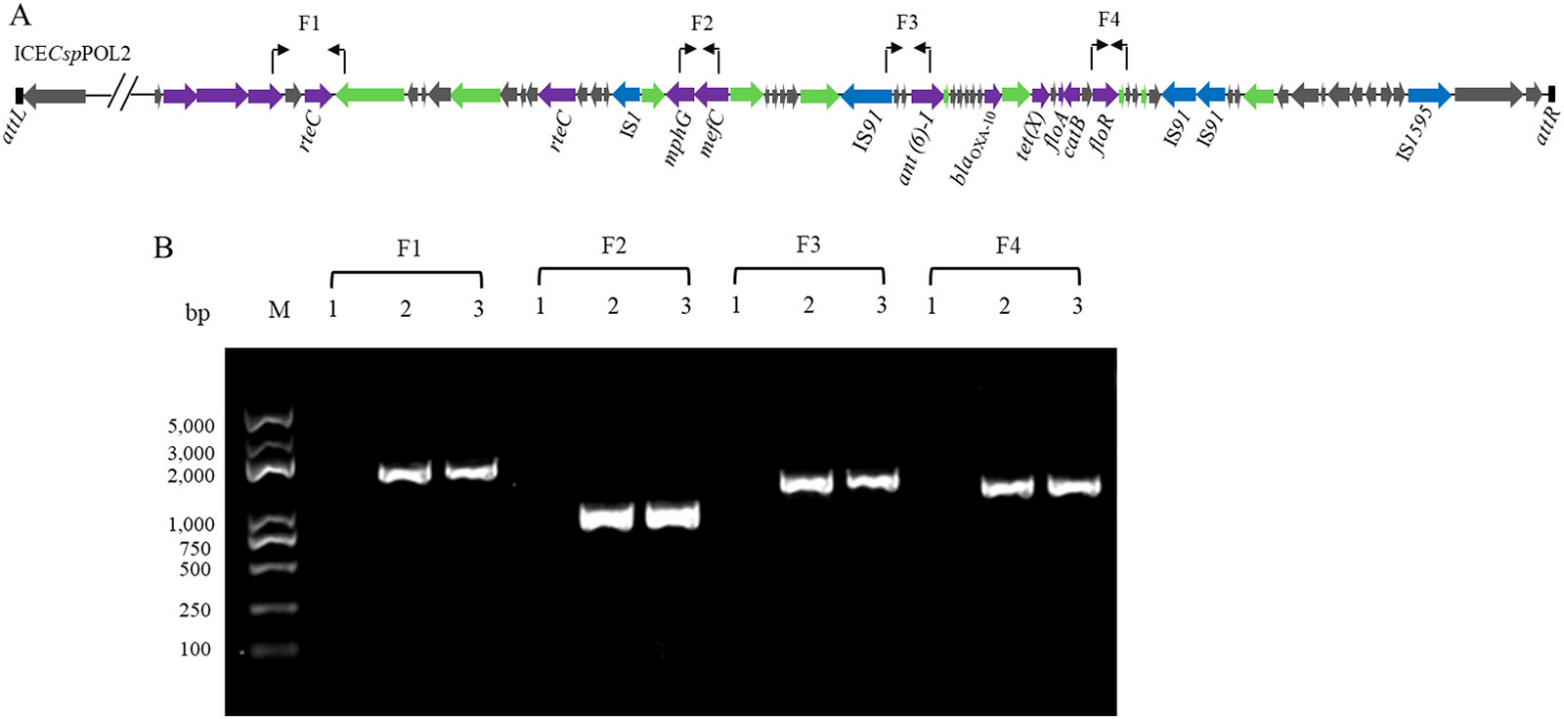
Verification of the presence of ICE*Csp*POL2. (A) Primer positions in ICE*Csp*POL2 are indicated using bent arrows. (B) Four ICE*Csp*POL2 fragments were amplified by PCR using total DNA of strain M6 (lane 1), transconjugant M6-P2 (lane 2), strain POL2 (lane 3) as the templates.

ICE*Csp*POL2 is a typical ICE, and the phylogenetic tree and BLASTn analysis revealed that it may have evolved from ICE*Ea*III of E. anophelis. To determine whether ICE*Csp*POL2 could be horizontally transferred among bacteria, conjugation assays were conducted using POL2 as the donor strain with different recipient strains. When *Elizabethkingia* sp. M6 was used as the recipient strain with meropenem (16 mg/liter) and sodium azide (220 mg/liter) as the selective pressure, the transconjugation frequency was about 1.02 × 10^−7^ colony forming units (CFU)/donor. One of the transconjugants was selected and named M6-P2. To test whether ICE*Csp*POL2 was inserted into the M6 genome, a PCR test and DNA sequencing were performed. The results revealed that the four chosen gene fragments of ICE*Csp*POL2 were present in the transconjugant M6-P2 but not in the recipient strain M6 (Fig. 6). Additionally, MIC analysis showed that the transconjugant *Elizabethkingia* sp. M6-P2 acquired resistance to antibiotics associated with ICE*Csp*POL2 resistance genes, especially meropenem (Table 1). The above data indicated that ICE*Csp*POL2 was horizontally transferred from the donor strain POL2 to the recipient strain *Elizabethkingia* sp. M6. However, when using the sodium azide-resistant Escherichia coli 25DN and E. coli DL21 as the recipient strains, no transconjugant was obtained.

## DISCUSSION

Vigilance against the spread of ARGs and antibiotic-resistant bacterial infections has become increasingly important. *Chryseobacterium* spp. and *Elizabethkingia* spp. are opportunistic pathogens belonging to the family *Flavobacteriaceae* (15, 16). *Chryseobacterium* spp. and *Elizabethkingia* spp. are reported to be resistant to multiple different antibiotics such as lactam antibiotics (imipenem, piperacillin, meropenem, ceftazidime, cefepime, cefpirome), aminoglycoside antibiotics (gentamicin, tobramycin, amikacin), and quinolone antibiotics (ciprofloxacin, levofloxacin) in many isolates (17–20). This resistance is of clinical concern as the acquisition of *Chryseobacterium* and *Elizabethkingia* infections in intensive care units has been increasing in recent years (12, 13). *Chryseobacterium* species are ubiquitous inhabitants of the water, soil, and hospital environments (21–23), whereas *Elizabethkingia* species have aroused much concern in recent years (12, 13) because they are associated with disease-carrying mosquitoes, including the dengue fever vector *Aedes* and the malaria vector *Anopheles* (24–28), which are associated with clinically significant infections and high mortality.

**FIG 7:**
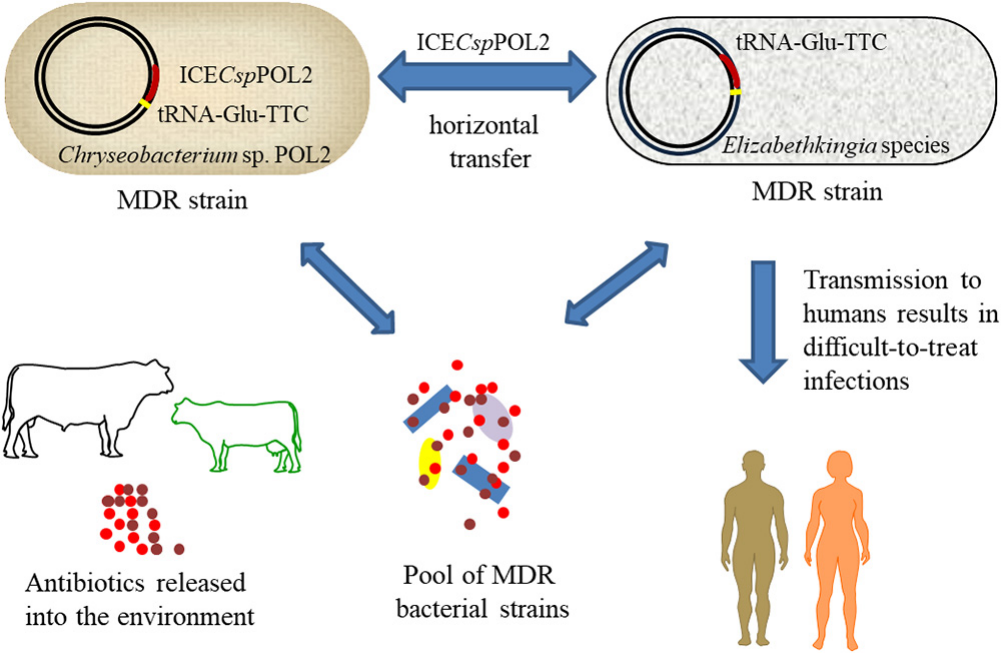
A simple model showing that ICE*Csp*POL2 mediated the dissemination of ARGs in the environment.

In this study, the multi-antibiotic-resistant *Chryseobacterium* sp. POL2 was isolated from livestock wastewater. POL2 had notable resistance to carbapenem antibiotics. An ICE named ICE*Csp*POL2 was identified in the *Chryseobacterium* sp. POL2 strain, and our bioinformatics analyses suggested that ICE*Csp*POL2 evolved from related ICE*Ea*s of E. anophelis strains. Further analysis indicated that ICE*Csp*POL2 could be horizontally transferred to *Elizabethkingia* sp. M6, a strain that was isolated from hospital wastewater. Because ICE*Csp*POL2 contains ARGs that could confer resistance to meropenem, acquisition of ICE*Csp*POL2 by environmental strains of *Elizabethkingia* could result in infections that would be difficult to treat. Reports have shown that ICE*Ea*s spread in *Elizabethkingia* spp., and that these ICEs mediate horizontal transfer of genes associated with antibiotic resistance, virulence, and stress response (12, 14, 27, 29–31). Therefore, these ICE*Ea*s can enable their bacterial hosts to quickly adapt to changing ecological niches and enable them to establish infections in humans. In this report, ICE*Csp*POL2 was found to disseminate multiple ARGs among *Chryseobacterium* and *Elizabethkingia* species (Fig. 7), highlighting the risks associated with the potential transmission of such ICEs in the environment, particularly with regard to their potential to cause outbreaks of multi-antibiotic resistant *Chryseobacterium* and *Elizabethkingia* species.

Bioinformatics analysis suggested that ICEs, rather than conjugative plasmids, are likely more important in the transmission of antimicrobial resistance in prokaryotes (8). The current NCBI genome database documents only five plasmids among the 261 genomes of *Chryseobacterium* species and two plasmids among the 202 genomes of *Elizabethkingia* species. In contrast, ICEs are commonly identified in the genomes of these two genera, such as the three types of ICEs (ICE*Ea*I, ICE*Ea*II, and ICE*Ea*III) that have been identified in E. anophelis strains isolated from around the world (14), suggesting that ICEs play an important role in horizontal gene transfer in these genera. *Chryseobacterium* sp. POL2, which was isolated in this study, contains only one chromosome, and as noted, ICE*Csp*POL2 in the POL2 genome is closely related to the ICE*Ea*s from *Elizabethkingia* species, suggesting that ICEs might contribute to the dissemination of antimicrobial resistance among *Chryseobacterium* spp. and *Elizabethkingia* spp.

Phylogenetic analysis showed that ICE*Csp*POL2 was most closely related to several members of the ICE*Ea*III group of E. anopheles ICEs. Some evidence suggests that ICEs of different groups favor different integration sites. ICE*Ea*I can integrate into a gene or intragenic regions, such as the *mutY* gene of the Wisconsin outbreak strain of E. anophelis (12, 14). ICE*Ea*II and ICE*Ea*III were found to insert next to tRNA genes, with ICE*Ea*II mainly inserted into *tRNA-Leu-CAA*, whereas ICE*Ea*III can insert into different tRNA genes, such as *tRNA-Glu-TTC*, *tRNA-Arg-ACG*, and *tRNA-Asp-GTC* (14). Analysis by ICEberg 2.0 verified that ICE*Csp*POL2 was inserted into the 3′ end of the *tRNA-Glu-TTC* gene of the *Chryseobacterium* sp. POL2 genome, and further analysis indicated that ICE*Csp*POL2 was also inserted into the 3′ end of the *tRNA-Glu-TTC* gene of the transconjugant *Elizabethkingia* sp. M6-P2 strain. Together with the phylogenetic analysis, this insertion pattern further suggests that ICE*Csp*POL2 belongs to the ICE*Ea*III group of ICEs.

Because ICE*Csp*POL2 encodes a type IV secretion/conjugation system, we tested whether this ICE could be horizontally transferred to other bacteria, such as Escherichia coli strains 25DN and DL21. However, we did not obtain transconjugants using these recipient strains, and further analysis found that both the *att* site (5′-ATTCCCCTACGGGCTACT-3′) and insertion site (*tRNA-Glu-TTC* gene) of ICE*Csp*POL2 do not exist in the 25DN genome (GenBank accession no. CP049298) or DL21 genome (GenBank accession no. CP079747). Further bioinformatics analysis with the *tRNA-Glu-TTC* gene sequence using BLASTn revealed that *tRNA-Glu-TTC* mainly exists in *Chryseobacterium* and *Elizabethkingia* species, indicating that ICE*Csp*POL2 may not have a broad host range and that *Chryseobacterium* and *Elizabethkingia* species may be the major hosts of ICE*Csp*POL2.

In conclusion, the integrative and conjugative element ICE*Csp*POL2, which was associated with the transmission of a carbapenem resistance gene, was identified in the genome of the multi-antibiotic-resistant strain *Chryseobacterium* sp. POL2. ICE*Csp*POL2 is closely related to the ICE*Ea*s from *Elizabethkingia* species, and conjugation assays found that ICE*Csp*POL2 can horizontally transfer to *Elizabethkingia* species, with the *tRNA-Glu-TTC* gene as the insertion site. Because *Elizabethkingia* species are associated with clinically significant infections and high mortality, the ability of ICE*Csp*POL2 to transfer carbapenem resistance from environmental strains of *Chryseobacterium* to *Elizabethkingia* is of clinical concern. Indeed, as ICE*Ea*s, including ICE*Csp*POL2, could contribute to the dissemination of multiple types of ARGs among *Chryseobacterium* and *Elizabethkingia* spp., it will be important to track the spread of ICE*Ea*s in hospital and environmental strains of *Chryseobacterium* and *Elizabethkingia* to prevent further outbreaks of MDR bacteria.

## MATERIALS AND METHODS

### Strains.

Strain POL2 was isolated from a wastewater sample from a livestock farm in Shandong, China. 10-fold serial dilutions of the wastewater sample were prepared with sterilized water, plated onto LB agar supplemented with 16 mg/liter tetracycline, and incubated overnight at 28°C to obtain single colonies. Next, a single colony was picked and streaked three consecutive times onto LB agar containing tetracycline to obtain a pure culture. A single colony was then selected and grown as a pure culture, which was named strain POL2.

The sodium azide-resistant strains Escherichia coli 25DN and DL21 were obtained from our laboratory stocks (32) and grown at 37°C in LB medium. *Elizabethkingia* sp. M6 strain, which was previously isolated from hospital wastewater, was stored in our laboratory before growing at 28°C in LB medium.

### Antibiotic susceptibility testing.

To determine the MICs of different antibiotics for strain POL2, antibiotic susceptibility testing was performed using the broth microdilution method (33). The recipient strain M6 and the transconjugant M6-P2 used in the conjugation assays below were also tested for MICs. All the antibiotic susceptibility tests in this study were carried out in triplicate.

### Whole-genome sequencing and genomic analysis.

The POL2 genome was sequenced using the Nanopore and BGISEQ-500 platform (BGI, Wuhan, China) and assembled using Unicycler software (34). Genome annotation was performed using the Prokaryotic Genome Annotation Pipeline (PGAP) on the NCBI website (https://www.ncbi.nlm.nih.gov/genome/annotation_prok/). Additional POL2 genome annotation was performed using the RASTtk server (35, 36) and the Pathosystems Resource Integration Center (PATRIC) server (37). The virulence factors in the POL2 genome were predicted using the Pathogen Host Interactions (PHI) database (38). Sequence alignment was performed using the BLAST server (https://blast.ncbi.nlm.nih.gov/Blast.cgi) and UniProt server (https://www.uniprot.org/blast/).

### Phylogenetic affiliation analysis of strain POL2.

To determine the taxonomic status of POL2, molecular phylogenetic analysis of *Chryseobacterium* sp. POL2 was analyzed based on its genome sequences. Twenty-seven genome sequences belonging to *Chryseobacterium* and *Elizabethkingia* species were selected, and the whole-genome phylogenetic tree was constructed using the PATRIC server (37).

### Identification of the integrative and conjugative element.

The ICE in the chromosome of strain POL2 was first predicted by IslandViewer 4 (39) and then further analyzed using ICEberg 2.0 software (40). The novel ICE was named ICE*Csp*POL2, and genes in ICE*Csp*POL2 were annotated using NCBI and the RASTtk server (35, 36). Insertion sequences in ICE*Csp*POL2 were predicted using IS-Finder (41).

### Evolutionary analysis of ICE*Csp*POL2.

Pairwise alignment of ICE*Csp*POL2 and other genetic elements was conducted using the BLAST search tool, and further alignment was conducted using BioXM 2.6 software. To analyze the phylogenetic affiliation, the whole nucleotide sequence of ICE*Csp*POL2 was first compared with other genetic elements using the BLAST website, and then nine nucleotide sequences belonging to the relevant ICEs were selected from the NCBI database. Evolutionary analyses were conducted in MEGA7 (42).

### Conjugation assays.

To verify the horizontal transferability of the ICE*Csp*POL2 among different bacteria, conjugation assays were performed as previously described with some modifications (32). E. coli strains 25DN and DL21 and the *Elizabethkingia* sp. strain M6 were used as the recipient strains, and *Chryseobacterium* sp. POL2 was used as the donor strain. After mixing POL2 and a recipient strain, the mixture was cultured on LB agar plates with meropenem (16 mg/liter), sodium azide, and X-Gluc (5-bromo-4-chloro-3-indolyl-beta-d-glucuronic acid) to screen the transconjugants. The presence of ICE*Csp*POL2 in the transconjugants was demonstrated by PCR, using primers listed in Table S1, and DNA sequencing.

### Data availability.

The complete sequence of the chromosome of *Chryseobacterium* sp. strain POL2 was deposited in GenBank under accession no. CP049298.
