# Supplementary material for: The Integrative and Conjugative Element ICECspPOL2 Contributes to the Outbreak of Multi-Antibiotic-Resistant Bacteria for Chryseobacterium Spp. and Elizabethkingia Spp
Source: Microbiol Spectr. 2021 Dec 22;9(3):e02005-21. doi: 10.1128/Spectrum.02005-21 (PMC8694125; doi:10.1128/Spectrum.02005-21)
Supplement: SUPPLEMENTAL FILE 1 — Supplemental material. Download SPECTRUM02005-21_Supp_1_seq12.pdf, PDF file, 0.01 MB [file spectrum02005-21_supp_1_seq12.pdf]

Supporting information

The integrative and conjugative element ICE*Csp*POL2 contributes to the outbreak of multi-antibiotic resistant bacteria for *Chryseobacterium* spp. and *Elizabethkingia* spp.

Jiafang Fu [a,b], Chuanqing Zhong [c], Yingping Zhou [c], Mengru Lu [b], Gongli Zong [a,b], Peipei Zhang [a,b], Moutai Cheng [b], Guangxiang Cao [a,b,*]

[a] Department of Epidemiology, the First Affiliated Hospital of Shandong First Medical University, Jinan 250117, China

[b] College of Biomedical Sciences, Shandong First Medical University & Shandong Academy of Medical Sciences, Jinan 250117, China

[c] School of Municipal and Environmental Engineering, Shandong Jianzhu University, Jinan 250101, China

* Corresponding author: Guangxiang Cao, Department of Epidemiology, the First Affiliated Hospital of Shandong First Medical University; College of Biomedical Sciences, Shandong First Medical University & Shandong Academy of Medical Sciences, Qingdao Road 6699, Jinan 250117, Shandong, P.R. China. Tel.: +86 531 59567322; Fax: +86 531 59567322

Table S1 Primers used in this study.

**Oligonucleotide**	**DNA Sequence (5’→3)**
F1-For	GGAAACAGCAGAGAACATTG
F1-Rev	CGACTAAGCGAACTCAAAAC
F2-For	AGTGCTTTCGCTAATGATGG
F2-Rev	GCTGGCTATTCTTTCACTCG
F3-For	ACTCTCCTGTCCTTCCTTAC
F3-Rev	GTTTTAGCCCACATTTCCTG
F4-For	TAAACAAAAACAACGCCCCG
F4-Rev	GTCAAAACCCAAATCGCAAG

Table S2 Genes annotated in ICE*Csp*POL2

start	stop	strand	gene	function
1772240	1767114	-		hypothetical protein
1772913	1772233	-		hypothetical protein
1775361	1772989	-		hypothetical protein
1776096	1775374	-		hypothetical protein
1776934	1776086	-		Peptidase, M23/M37 family
1778918	1776924	-	*T4CP*	Putative mobilization protein BF0133
1779164	1778925	-		hypothetical protein
1780036	1779167	-	*traN*	Conjugative transposon protein TraN
1781253	1780039	-	*traM*	Conjugative transposon protein TraM
1781596	1781243	-		hypothetical protein
1782213	1781599	-	*traK*	Conjugative transposon protein TraK
1783469	1782219	-	*traJ*	Conjugative transposon protein TraJ
1784302	1783472	-		hypothetical protein
1784972	1784307	-		hypothetical protein
1785682	1784975	-		hypothetical protein
1786364	1785726	-		hypothetical protein
1789509	1786435	-	*traG*	Conjugative transposon protein TraG
1789795	1789481	-	*traF*	Conjugative transposon protein TraF
1790120	1789797	-	*traE*	Conjugative transposon protein TraE
1790690	1790157	-		hypothetical protein
1791406	1790753	-	*traA*	Conjugative transposon protein TraA
1792878	1791409	-		Putative conjugative transposon mobilization protein BF0132
1793225	1792875	-		hypothetical protein
1794043	1793249	-		hypothetical protein
1795264	1796514	+	IS*Lre2*	Integrase
1796773	1796654	-		hypothetical protein
1797044	1798117	+		Mycobacteriophage Barnyard protein gp56
1798170	1798580	+		hypothetical protein
1798583	1799098	+		hypothetical protein
1799735	1799352	-		hypothetical protein
1800818	1800045	-		hypothetical protein
1800896	1802035	+	*dinB*	DNA polymerase IV (EC 2.7.7.7)
1802050	1804989	+		Error-prone repair homolog of DNA polymerase III alpha subunit (EC 2.7.7.7)
1805568	1805257	-		hypothetical protein
1805875	1805744	-		hypothetical protein
1806594	1806145	-	*traQ*	Conjugative transposon protein TraQ
1807166	1806606	-	*traO*	Conjugative transposon protein TraO
1808090	1807188	-	*traN*	Conjugative transposon protein TraN
1809452	1808115	-	*traM*	Conjugative transposon protein TraM
1809726	1809439	-	*traL*	Conjugative transposon protein TraL
1810184	1809747	-		FIG01092846: hypothetical protein

1810818	1810195	-	*traK*	Conjugative transposon protein TraK
1811840	1810845	-	*traJ*	Conjugative transposon protein TraJ
1812475	1811843	-	*traI*	Conjugative transposon protein TraI
1815004	1812503	-	*traG*	Conjugative transposon protein TraG
1815333	1815001	-	*traF*	Conjugative transposon protein TraF
1815650	1815345	-	*traE*	Conjugative transposon protein TraE
1816484	1815858	-	*traD*	Conjugative transposon protein TraD
1817210	1816495	-	*traB*	Conjugative transposon protein TraB
1817989	1817222	-	*traA*	Conjugative transposon protein TraA
1818186	1818064	-		hypothetical protein
1818698	1819162	+		hypothetical protein clusted with conjugative transposons, BF0131
1819137	1820426	+		Putative conjugative transposon mobilization protein BF0132
1820529	1822529	+	*T4CP*	Putative mobilization protein BF0133
1823489	1822593	-		Transcriptional regulator, AraC family
1824470	1823607	-		hypothetical protein
1824602	1825051	+		hypothetical protein
1825056	1825238	+		hypothetical protein
1825861	1827216	+		Efflux transport system, outer membrane factor (OMF) lipoprotein
1827231	1830395	+		RND multidrug efflux transporter; Acriflavin resistance protein
1830398	1831495	+		RND efflux system, membrane fusion protein
1831498	1832220	+		hypothetical protein
1832336	1833163	+	*rteC*	Tetracycline resistance element mobilization regulatory protein RteC
1838648	1833216	-		Putative DNA methylase
1839072	1838635	-		hypothetical protein
1839245	1839421	+		Uncharacterized protein CHU_1311
1840339	1839458	-		FIG01093033: hypothetical protein
1842422	1840329	-		DNA topoisomerase III, Bacteroidales-type (EC 5.99.1.2)
1843906	1842425	-		FIG01092183: hypothetical protein
1844283	1844002	-		hypothetical protein
1844662	1844309	-		hypothetical protein
1845772	1844927	-	*rteC*	Tetracycline resistance element mobilization regulatory protein RteC
1846791	1846135	-		HlyD family secretion protein
1847438	1846800	-		hypothetical protein
1848056	1848526	+		hypothetical protein
1849627	1848557	-	IS*1*	Integron integrase
1849820	1850763	+		repeat region
1850802	1849918	-	*mphG*	Macrolide 2'-phosphotransferase => Mph(E)/Mph(G) family
1852030	1850807	-	*mefC*	Macrolide resistance, MFS efflux pump => Mef(C)
1850825	1852375	+		repeat region
1852392	1852240	-		hypothetical protein

1852407	1852985	+		hypothetical protein
1852987	1853643	+		hypothetical protein
1853640	1854626	+		Rossmann fold nucleotide-binding protein Smf possibly involved in DNA uptake
1854640	1856412	+		repeat region
1856321	1854747	-	IS*91*	Mobile element protein
1856645	1856752	+		hypothetical protein
1856777	1857025	+		hypothetical protein
1857156	1858118	+	*ant(6)-I*	Aminoglycoside 6-nucleotidyltransferase (EC 2.7.7.-) => ANT(6)-I
1858130	1858254	+		repeat region
1858406	1859176	+		hypothetical protein
1859551	1860132	+		hypothetical protein
1860340	1860218	-		hypothetical protein
1860380	1860844	+		hypothetical protein
1861444	1861866	+		Acetyltransferase, GNAT family
1862061	1862918	+	*bla*$_{OXA-10}$	Class D beta-lactamase OXA-10
1863220	1865483	+		repeat region
1863237	1863497	+	*tet (X)*	Tetracycline resistance, tetracycline-inactivating enzyme => Tet(X)
1863499	1864008	+		hypothetical protein
1864005	1864508	+	*floA*	Dihydrofolate reductase (EC 1.5.1.3)
1865341	1864712	-	*catB*	Chloramphenicol O-acetyltransferase (EC 2.3.1.28) => CatB family
1865582	1866496	+		Hydrolase, alpha/beta fold family
1866489	1867712	+	*floR*	Chloramphenicol/florfenicol resistance, MFS efflux pump => FloR family
1867821	1868010	+		repeat region
1868048	1868554	+		hypothetical protein
1868551	1868733	+		hypothetical protein
1868572	1868696	+		repeat region
1868746	1869597	+		hypothetical protein
1870998	1869820	-	IS*91*	Transposase
1871771	1870983	-	IS*91*	integrase family protein
1872196	1872098	-		hypothetical protein
1872501	1872629	+		hypothetical protein
1873570	1872731	-		DNA primase, TraP-type
1873922	1873686	-		hypothetical protein
1875110	1873986	-		hypothetical protein
1875388	1875161	-		hypothetical protein
1876150	1875440	-		hypothetical protein
1876404	1876153	-		hypothetical protein
1876688	1876404	-		hypothetical protein
1876823	1877272	+		hypothetical protein
1877275	1877964	+		hypothetical protein
1877979	1879247	+	IS*1595*	Integrase

1879321	1882386	+	hypothetical protein
1882379	1883110	+	hypothetical protein
